# Giant Left Atrial Myxoma Causing Mitral Valve Obstruction and Concomitant Coronary Artery Disease

**DOI:** 10.4021/cr84w

**Published:** 2011-09-20

**Authors:** Turgut Karabag, Sait M. Dogan, Mustafa Aydin, Muhammet R. Sayin

**Affiliations:** a Zonguldak Karaelmas University, Faculty of Medicine, Department of Cardiology, Zonguldak, Turkey

**Keywords:** Myxoma, Coronary disease, Mitral valve obstruction, Coronary angiography

## Abstract

We describe a 56-year old male patient who presented with congestive heart failure and had undergone echocardiography that showed a mobile, giant left atrial mass which caused obstruction in the left ventricular outflow tract. The patient underwent a coronary angiography before operation that showed severe stenosis in the left anterior descending and circumflex artery. The patient underwent resection of the mass and coronary artery bypass grafting. The pathology of the mass was myxoma and patient had a satisfactory outcome.

## Introduction

Myxomas are the most common primary intracavitary tumors of the heart [1]. Approximately, 75% of myxomas originate in the left atrium in adults [2]. The symptoms of myxomas may mimic various cardiac and noncardiac conditions. Though rarely reported in the literature, myxomas are known to be accompanied by coronary artery disease or other valvular pathologies [3]. We herein present a case of coronary artery disease accompanied by giant left atrial myxoma in a 56-year old male patient presenting with heart failure symptoms.

## Case Report

**Figure 1:**
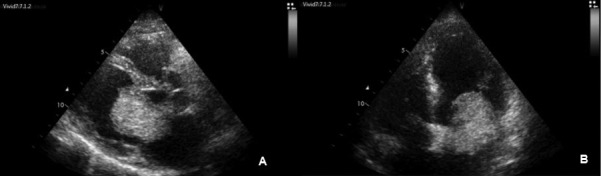
Echocardiography showing giant left atrial myxoma on parasternal long axis (A) and apical 4 chamber (B) imaging.

**Figure 2:**
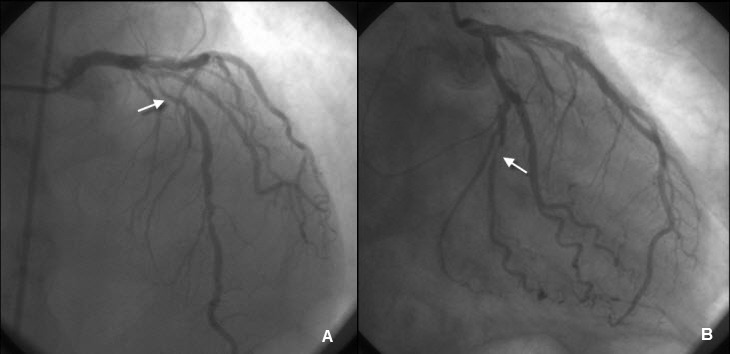
Coronary angiography showing critical stenosis (arrows) in left anterior descending (A) and circumflex (B) arteries.

A 56 year old male patient was admitted to our clinic with complaints of shortness of breath ongoing for three months. He reported that the severity and frequency of his complaints had increased recently. On admission, the patient was orthopneic and dyspneic and described a feeling of constriction in the chest. He had no other atherosclerotic risk factors except for tobacco abuse. On physical examination, blood pressure was 90/70 mmHg and pulse was 130/min. Bilateral crepitant rales were heard along the lungs. The patient had 1 + edema in both legs. Cardiac oscultation revealed 1/4 diastolic rulman and a 2/6 systolic murmur at the apex and at the mitral focus. Electrocardiography (ECG) revealed T wave negativity on chest derivations C4-6. A venous blood sample showed hypercholesterolemia and a sedimentation rate of 81 mm/hour. Based on these findings, transthoracic echocardiography was performed and revealed a 44 x 31 mm mass in the left atrium. The mass was arising from the atrial side of the anterior mitral valve and was moving in and out of the left ventricle with each cardiac cycle and causing obstruction of the mitral valve (Fig. 1A, B). Global systolic function was normal. Both atria were enlarged and pulmonary artery systolic pressure was 48 mmHg. Surgical removal of the mass was decided due to the size of the mass, stenosis in the left ventricular inflow tract and high risk for thromboembolism and sudden death. Based on the patient’s complaints, risk factors and ischemic ECG findings, preoperative coronary angiography was decided. Coronary angiography showed critical stenosis of the left anterior descending artery (LAD) and circumflex artery (LCx) and noncritical stenosis of the right coronary artery (RCA) (Fig. 2A, B). Coronary angiography showed no findings suggestive of tumor vascularity. Ventriculography was not performed due to the potential risk of embolism. The patient was advised to undergo surgery for the resection of the left atrial mass and coronary bypass surgery. The mass arising from the mitral valve was resected and LIMA-LAD and SVG-OM1 bypass grafting was performed. The pathology report was consistent with myxoma. An echocardiography, performed at follow-up examination 2 months postoperatively, revealed no evidence of residual mass. Minimal mitral regurgitation was present. Pulmonary artery systolic pressure decreased to 38 mmHg. The patient had no complaints and his functional capacity was NYHA class 1-2. His ECG was completely normal.

## Discussion

Cardiac myxomas are the most common benign tumors of the heart in adults and account for 25% of all cardiac tumors and 50% of benign tumors [1]. They are ususally mobile pedunculated masses originating from the fossa ovalis. It was reported that 20% of all myxomas occurred in the right atrium [2]. The clinical presentation of patients with myxomas varies according to the tumor location, site and mobility [4]. Transthoracic echocardiography is the primary modality for the diagnosis of myxoma [5]. The most common symptoms include dyspnea, atypical chest pain and obstructive and embolic phenomena [6]. Myxomas can cause a wide range of nonspecific clinical findings and symptoms. Patients with myxomas may present with chest pain, dyspnea and syncope. These symptoms may also occur in patients with critical coronary artery stenosis. The mean age of patients with myxoma is 56 years of age during which coronary artery disease is common and 70% are females [3]. Coronary artery disease should be suspected and investigated in patients with myxoma since the age at presentation and the symptoms can be similar in patients with myxoma and those with coronary artery disease [7]. Even though performing preoperative coronary angiography in patients who are planned to undergo surgery remains controversial, routine coronary angiography is recommended in patients with a history of angina and those above 35 years of age [5]. Myxomas are rare tumors but surgical treatment is an effective intervention [8]. Recurrence may occur in 3% of sporadic cases and 20% of familial cases after resection [9, 10].

In the case presented in this study, an emergency operation was decided upon due to the size and location of the mass and the deterioration in the patient’s hemodynamics. However, considering history of smoking and the presence of atherosclerotic risk factors such as hyperlipidemia and concomitant ischemic ECG changes, we decided to perform preoperative coronary angiography. The patient’s complaints improved after succesful surgical treatment. Echocardiography performed at three months postoperatively revealed no evidence of recurrence.

In conclusion, surgery should be considered as soon as the diagnosis of myxoma is established and the presence of concomitant conditions such as coronary artery disease should be taken into consideration before surgery. Coronary angiography should be performed particularly in middle aged or elderly patients before surgery.

## References

[1] Peters PJ, Reinhardt S (2006). The echocardiographic evaluation of intracardiac masses: a review. J Am Soc Echocardiogr.

[2] Murphy DP, Glazier DB, Krause TJ (1997). Mitral valve myxoma. Ann Thorac Surg.

[3] Sankar NM, Vaidyanathan RK, Prasad GN, Cherian KM (2006). Left atrial myxoma presenting as acute inferior wall infarction-a case report. J Card Surg.

[4] Dujardin KS, Click RL, Oh JK (2000). The role of intraoperative transesophageal echocardiography in patients undergoing cardiac mass removal. J Am Soc Echocardiogr.

[5] Erdil N, Ates S, Cetin L, Demirkilic U, Sener E, Tatar H (2003). Frequency of left atrial myxoma with concomitant coronary artery disease. Surg Today.

[6] Li AH, Liau CS, Wu CC, Chien KL, Ho YL, Huang CH, Chen MF (1999). Role of coronary angiography in myxoma patients: a 14-year experience in one medical center. Cardiology.

[7] Kejriwal NK, Tan J, Ullal RR, Alvarez JM (2003). Atrial myxoma with coexistent coronary artery disease: a report of two cases. Heart Lung Circ.

[8] Yavuz T, Peker O, Ocal A, Ibrisim E (2005). Left atrial myxoma associated with acute myocardial infarction. Int J Cardiovasc Imaging.

[9] van Gelder HM, O'Brien DJ, Staples ED, Alexander JA (1992). Familial cardiac myxoma. Ann Thorac Surg.

[10] Keeling IM, Oberwalder P, Anelli-Monti M, Schuchlenz H, Demel U, Tilz GP, Rehak P (2002). Cardiac myxomas: 24 years of experience in 49 patients. Eur J Cardiothorac Surg.

